# A Machine Learning-Based Approach for Predicting Patient Punctuality in Ambulatory Care Centers

**DOI:** 10.3390/ijerph17103703

**Published:** 2020-05-24

**Authors:** Sharan Srinivas

**Affiliations:** Department of Industrial and Manufacturing Systems Engineering, College of Engineering, and Department of Marketing, Trulaske College of Business, University of Missouri, Columbia, MO 65211, USA; srinivassh@missouri.edu

**Keywords:** machine learning, late-arriving patients, clinical decision support, ambulatory care center, predicting tardy arrivals

## Abstract

Late-arriving patients have become a prominent concern in several ambulatory care clinics across the globe. Accommodating them could lead to detrimental ramifications such as schedule disruption and increased waiting time for forthcoming patients, which, in turn, could lead to patient dissatisfaction, reduced care quality, and physician burnout. However, rescheduling late arrivals could delay access to care. This paper aims to predict the patient-specific risk of late arrival using machine learning (ML) models. Data from two different ambulatory care facilities are extracted, and a comprehensive list of predictor variables is identified or derived from the electronic medical records. A comparative analysis of four ML algorithms (logistic regression, random forests, gradient boosting machine, and artificial neural networks) that differ in their training mechanism is conducted. The results indicate that ML algorithms can accurately predict patient lateness, but a single model cannot perform best with respect to predictive performance, training time, and interpretability. Prior history of late arrivals, age, and afternoon appointments are identified as critical predictors by all the models. The ML-based approach presented in this research can serve as a decision support tool and could be integrated into the appointment system for effectively managing and mitigating tardy arrivals.

## 1. Introduction

Late patient arrival is widespread and a prominent concern in several ambulatory clinics across the globe [1,2,3]. The reported rates of prevalence varied substantially among specialties, such as 10% in pediatrics [4], 22% in urology [2], and 38% in cardiology [5]. Clinics serving tardy arrivals may experience detrimental ramifications such as schedule disruption, ineffective doctor utilization, and increased waiting time for forthcoming patients who may have arrived on-time [6]. This would, in turn, lead to severe repercussions such as an imbalanced physician workload, higher health care costs, and compromised service quality [3]. On the other hand, refusing treatments for late arrivals could pose severe health and malpractice risks. 

Realizing the adverse consequences, many studies have focused on mitigating late arrivals using operational strategies such as prioritizing on-time arrivals [7], instituting rescheduling policies [8], and sending automated text message reminders [9]. While these measures can be beneficial, the clinic may still experience substantial tardy arrivals. To effectively manage unpunctual patients and surmount the adverse effects, a medical center must adopt targeted intervention strategies and develop smart scheduling policies that integrate a patient’s late arrival risk. This is only possible if the clinic can identify the patients who are likely to be tardy for their appointment in advance. Given the significance of early detection, numerous efforts have been taken to predict the patient-specific risk of late arrivals [4,10,11,12]. Evidence suggests both patient-level (insurance type, patient’s primary language, and age) and visit-level (first-time patients, appointment time, day of the week) attributes to be associated with late arrivals [4,10,11,12].

Nevertheless, most existing models for predicting late arrivals have adopted traditional methods (different forms of regression analysis), limited predictor variables, and small samples. Moreover, these models are evaluated only based on their predictive power and tested on a single clinic. In this research, it is hypothesized that the use of machine learning (ML) algorithms and a large dataset with a comprehensive set of predictors can capture the complex synergistic interaction of risk factors to predict the patient-specific tardiness risk accurately. Two ambulatory care centers, an ear-nose-throat (ENT) clinic, and a women’s health (WH) clinic, experiencing a high incidence of tardy arrivals, are considered. Further, their retrospective electronic medical record (EMR) data are leveraged for model building. Since each ML algorithm may differ in its training approach, the study aims to (i) conduct a comparative analysis of four ML models (logistic regression, random forests, gradient boosting machine, and artificial neural networks), with respect to predictive performance, computational time and interpretability, and (ii) identify model-specific critical predictors for patient punctuality. 

## 2. Materials and Methods

**Figure 1:**
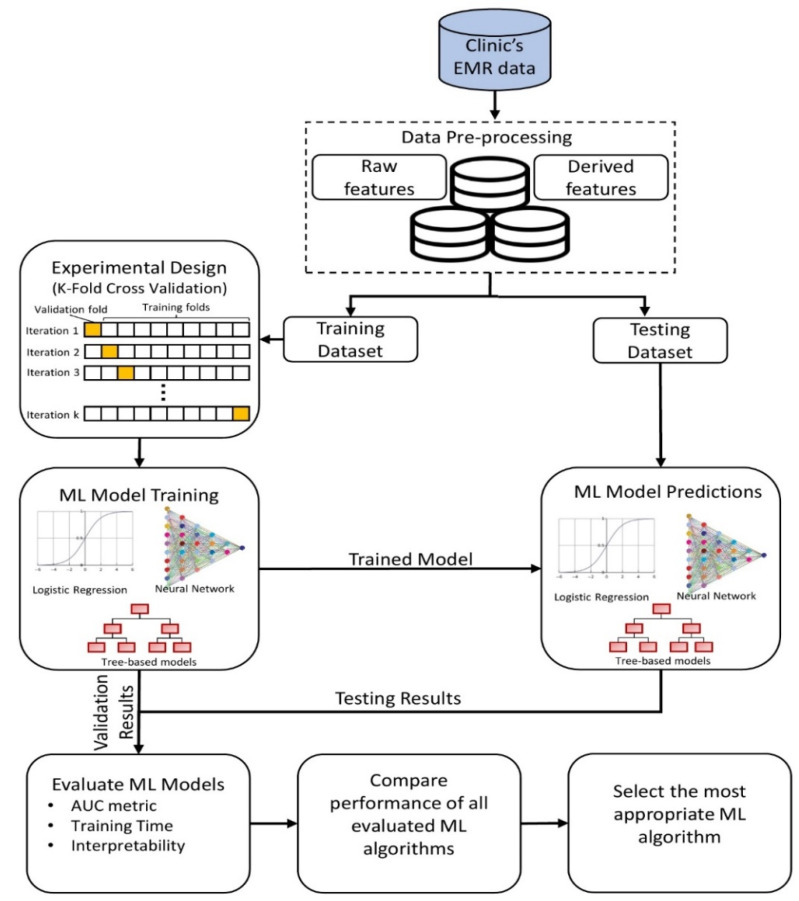
A methodology framework for machine learning-based prediction of late arrivals.

In this research, a systematic and structured approach is adopted for (i) pre-processing the EMR data, (ii) designing the experiment for training different ML models, (iii) evaluating ML models, and (iv) deploying a suitable model for informing the practitioners in advance (Figure 1).

### 2.1. Data Collection and Pre-Processing

The data for this research are obtained from two different specialty clinics (ENT and WH) located at a regional medical center in Pennsylvania, USA. For each clinic, the last two years of EMR data are extracted upon obtaining approval from the institutional review board. During this period, the ENT facility and WH clinic had 46,421 and 78,294 patient visits, respectively. Each record extracted includes the following information:

- Patient characteristics—age, gender, race, marital status, insurance type, patient type (new vs. return), medical record number (MRN), and zip code;
- Visit information—appointment duration, appointment time, appointment date, timestamps of patient arrival time, treatment begin time, and check-out time.

In consultation with the clinical care team, 21 predictor variables are identified, where each of them can be grouped into one of the four categories: patient-level (could change for every patient), appointment-level (could differ for every visit), clinic-level (could vary for each clinic), and environment-related (could fluctuate for every hour of the day), as shown in Table 1. Moreover, the predictor variables employed can be a field extracted from the EMR (raw feature) or derived from one or more fields available in the EMR (derived feature). 

While the raw features are directly used in the prediction algorithm, the derived features are pre-processed in multiple ways. The predictor variable “Visit Count”, which denotes the rolling sum of the number of visits by a patient, is computed based on the MRN (a unique identifier for each patient) extracted from the EMR. The “Commuting Distance” for each patient is obtained by leveraging an application programming interface (API) for Google Maps, which provides the driving distance between the patient’s and clinic’s zip codes. Another derived patient-related feature, neighborhood socioeconomic status (SES), is a composite score obtained by combining the normalized value of the average household income and education level corresponding to the patient’s zip code. The appointment-level derived feature “Lateness History”, a patient’s rolling history of late arrivals for a given appointment, is determined by adding the number of times that patient (identified by MRN) was tardy in the past. The other appointment-level derived feature indicates whether a visit is scheduled before or after a national holiday. This information is determined by comparing the appointment date to the list of national holidays in the USA. The hourly climate forecast (environment-related variables) on the day of the appointment, namely, temperature, visibility, and weather conditions, are derived using the API for a commercial weather service provider. 

The outcome variable is dichotomous—on-time and late arrival. It is determined for each patient using the EMR timestamp data. A patient is considered to be tardy if the difference between the actual arrival time and scheduled check-in time is over five minutes and punctual otherwise. The reason for allowing a five-minute grace period is that some patients may arrive on-time at the clinic but are checked-in a few minutes late if the front desk staff is busy. Moreover, the clinic can accommodate patients coming up to five minutes past the scheduled appointment time without incurring a substantial disruption. The distribution of predictor variables that are common to both the clinics is shown in Table 2. 

### 2.2. Predictive Modeling

The goal of any predictive model is to uncover the function that describes the relationship between the features and outcome variable based on representative training examples (historical cases). To prepare the data for predictive modeling and evaluation, they are randomly split into two parts: training and testing. Subsequently, the 21 features and the corresponding outcome from the training dataset are presented as examples to the ML model. Besides, to avoid the risk of overfitting (or learning the noise), a *k*-fold cross-validation procedure is performed in the learning phase, where the training dataset is divided into *k* subsets in which one subset is used for validation, and the remaining subsets are used for learning the examples. The process is repeated until each of the subsets is used exactly once for validation. Finally, the trained model, which has hypothesized a function between the predictors and outcome, is used to predict the risk of late arrival only based on the features from the testing dataset (unseen examples). 

While the model building and evaluation procedure are consistent across different supervised learning algorithms, each classification model may adopt a unique approach to learn the relationship between the features and outcome, which, in turn, affects the predictive performance, time required to train, and ability to explain the mapping function. Further, it is difficult to identify the most appropriate algorithm for a given dataset. Therefore, in this research, a comparative analysis of four popular ML algorithms that are diverse with respect to their training approach is conducted: logistic regression (LR) [13], feed-forward artificial neural network with backpropagation learning (ANN) [14], random forest (RF) [15], and stochastic gradient boosting machines (GBM) [16]. The hyperparameters of the ML algorithms are tuned using a grid search method, which performs an exhaustive search through a user-specified parameter space during training and returns the best parameter in that space. Mainly, the reason for examining these four algorithms is as follows. The LR algorithm is one of the most widely used algorithms for classification tasks in the medical domain as it is easy to understand and performs well for linearly separable datasets [17,18,19]. Recently, ANN has also played a significant role in medical decision support as it is routinely used to detect anomalies in clinical systems, obtain diagnostic/prognostic inferences, and gain insights on health outcomes [17,20]. However, ANN is sensitive to changes in the training data, which could lead to high variance in the predictions [21]. Ensemble methods, which combine the predictions of multiple algorithms, are known to reduce the variance and yield a superior predictive performance [22]. In particular, tree-ensembles (GBM and RF) have consistently outperformed other ML algorithms in the literature [23,24]. 

#### 2.2.1. Logistic Regression

LR is a statistical learning model that represents the binary outcome variable (i.e., on-time or late arrivals) using a sigmoid function with a weighted linear combination of the predictor variables [13]. Thus, the predicted probability of a certain outcome (e.g., late arrival) can be expressed as shown in Equation (1), where $X_{i}$ are the independent variables (e.g., $X_{1}$ = Age, $X_{2}$ = Sex, …, $X_{19}$ = Weather Condition) and $\beta_{i}$ are the weights as determined by the maximum likelihood method.

(1) $$P\left(Y=late\;arrival\right)=\frac{e^{\left(\beta_{0}+\beta_{1}X_{1}+\beta_{2}X_{2}+\ldots +\beta_{N}X_{N}\right)}}{1+e^{\left(\beta_{0}+\beta_{1}X_{1}+\beta_{2}X_{2}+\ldots +\beta_{N}X_{N}\right)}}$$

#### 2.2.2. Feed-Forward Artificial Neural Networks with Backpropagation Learning 

The artificial neural network [14] bases its algorithm on the biological neural network to learn the relationship between predictors and an outcome. While there are different types of neural networks, a feed-forward artificial neural network with backpropagation learning (ANN) is used in this research as it is most suitable for the classification task under study. The ANN consists of three interconnected layers (input, hidden, output), where each layer has a specified number of nodes. Each node (*j*) at a given layer (*l*) is connected to a node (*i*) in the next layer by a connection weight (*w_ij_*). Further, each predictor corresponds to a node in the input layer. The ANN uses a forward and backward pass as a learning mechanism. The forward pass is used to predict the outcome ($\hat{Y}$) from the input variables through the hidden layer(s) by calculating the weighted sum of values incident at each node. The activity or output at node *i* ($o_{i}$) during the forward pass is calculated as shown in Equation (2), where $v_{j}$ denotes the value of node *j* in the previous layer. After each iteration (*k*) of the forward pass, a backward pass is performed to progressively alter (if required) the weights for the next iteration (*k* + 1), such that the squared difference between the predicted and expected outcome is minimized (Equation (3)). The weights for iteration, *k* + 1, is adjusted as shown in Equation (4), where ρ denotes the learning rate. Both the forward and backward passes are repeated until the model is fully trained (i.e., weights are optimized). The parameters for the ANN considered in this research are the number of nodes in the hidden layer (*H*) and the learning rate (ρ).

(2) $$o_{i}=\frac{1}{1+e^{-\left(w_{0}+\sum_{j}w_{ij}v_{j}\right)}}$$

(3) $$E={\left(Y-\hat{Y}\right)}^{2}$$

(4) $$w_{ij}^{k+1}=w_{ij}^{k}-\rho {\left(\frac{\partial E}{\partial w_{ij}}\right)}^{k}$$

#### 2.2.3. Tree-based Ensemble Methods

RF [15] and GBM [16] are decision-tree-based algorithms, which use an ensemble of classification trees for predicting a categorical outcome. Unlike logistic regression, a classification tree is a non-parametric method that recursively partitions data into two subsets based on a chosen independent variable *X_i_* with a spilt-point *c (*$X_{i}^{c}$*)*. The algorithm begins with a root node, where a binary partition (*M_1_* and *M_2_*) of the *M* training samples is performed based on the split-point of predictor *X_i_* (i.e., *M_1_ = {***X**| *X_i_ ≤ c}* and *M_2_ = {***X**| *X_i_ > c}*). To choose the best feature for partitioning at each iteration, Gini gain ($\hat{\Delta G}\left(M,\;X_{i}^{c}\right)$), a measure for evaluating the split of *M* samples based on $X_{i}^{c}$, is used (Equation (5)). Note that $\hat{G}\left(M\right)$ in Equation (5) is equivalent to $1-\underset{j}{\sum }{\left(p_{j}\right)}^{2}$, where $p_{j}$ denotes the proportion of samples belonging to outcome class j ϵ {on-time arrival, late arrival} in *M* examples.

(5) $$\hat{\Delta G}\left(M,\;X_{i}^{c}\right)=\hat{G}\left(M\right)-\frac{\left|M_{1}\right|}{\left|M\right|}\hat{G}\left(M_{1}\right)-\frac{\left|M_{2}\right|}{\left|M\right|}\hat{G}\left(M_{2}\right)$$

The best split-point *c* for variable $X_{i}$ is obtained using Equation (6), and the best feature for splitting is then determined based on Equation (7).

(6) $${\hat{\Delta G}}_{i}^{max}=\underset{c}{\max}\hat{\Delta G}\left(M,\;X_{i}^{c}\right)$$

(7) $$X_{i}{}^{*}=X_{\underset{i=1,2,\ldots ,N}{\mathrm{argmax}\left({\hat{\Delta G}}_{i}^{max}\right)}}$$

Likewise, each new node is split based on a chosen feature ${\left\{X_{i}\right\}}_{i=1}^{N}$, resulting in a tree-like structure. The procedure is repeated until a stopping criterion is met (e.g., no change in Gini gain after splitting). 

The RF algorithm builds *T* classification trees, where each tree only uses a bootstrapped subset of the *M* training examples. However, unlike the traditional decision tree algorithm, the RF algorithm chooses the best feature split among the randomly selected *m (< N)* predictors at each node. Upon training, the final prediction is the majority vote of all *T* classification trees. In GBM, a shallow (weak) classification tree is fit at each iteration on a random subsample of the training data (selected without replacement). The depth of each classification tree is controlled by two parameters: number of splits in each tree (*S*) and minimum samples required in a terminal node (*R*). The GBM algorithm aims to achieve incremental improvement in subsequent iterations by prioritizing training samples that were incorrectly classified in the previous iterations. A learning rate (ρ) is specified to emphasize the importance of rectifying the errors of the prior models. Finally, a weighted vote of all the classification trees is used to predict the outcome category. Thus, GBM adopts an additive training approach by sequentially fitting *T* classification trees.

### 2.3. Model Evaluation

The ML models are evaluated based on three criteria: predictive performance, computational complexity, and interpretability. The area under the receiver operating characteristic (AUC) value, which ranges between 0 and 1, is used to assess the predictive performance. An AUC score of 1 indicates a perfect classification performance, while an AUC score of 0.5 represents a model that is equivalent to a random guess. Typically, a classifier achieving an AUC value of 0.8 or higher is regarded to be a good model [19,25]. Besides, the statistical significance between the AUC values of ML algorithms is established using the DeLong’s method [26]. Computational complexity is estimated based on the time required to train an ML algorithm. Finally, interpretability is evaluated based on an algorithm’s ability to identify the key predictors and explain its influence on patient punctuality. 

## 3. Results

The ENT facility had 22% of late-arriving patients, while the WH clinic had a comparatively higher incidence of unpunctual arrivals (32.8%). About 70% of EMR data (32,495 ENT visits and 54,806 WH visits) is used for training the ML algorithms using a 10-fold cross-validation procedure. The analysis is performed with R version 3.2.3 using the caret package for ML model development/analysis [27] and the pROC package for comparing the AUC values using Delong’s method [28]. All the analyses were executed on a computer running the Intel Core i7 4.20 GHz processor, Windows 10 operating system, and 64 GB RAM.

**Figure 2:**
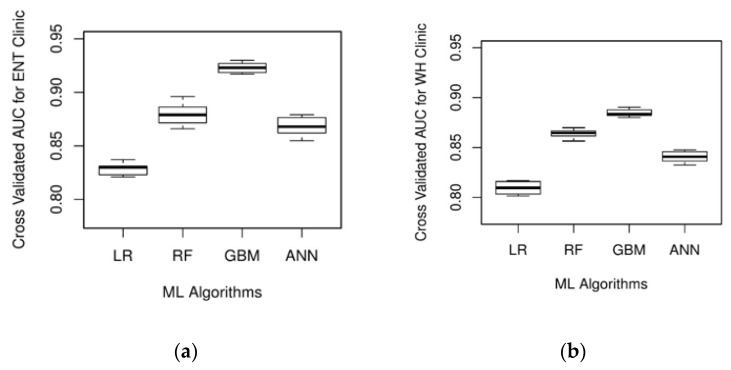
Box plot of area under the receiver operating characteristic (AUC) values based on 10-fold cross-validation for (**a**) ENT clinic and (**b**) WH clinic.

The ML algorithm’s prediction on the cross-validation and testing dataset is used to compute the AUC values and appraise its performance. It can be observed from Figure 2 that all the ML algorithms have an average cross-validated AUC value of 0.8 or above, indicating good discriminating ability. GBM produced the best result with an average cross-validated AUC value of 0.923 and 0.885 for ENT and WH clinics, respectively. Further, RF is the next best model that demonstrates superior performance. While GBM yields a significantly better AUC value than RF for the ENT clinic (*p*-value = 0.03), this metric is not significantly different for the WH clinic (*p*-value = 0.20). The predictive performance of GBM is also significantly higher than the ANN for the ENT and WH clinics. On the other hand, LR resulted in the least average AUC value on the validation dataset (AUC_ENT_ = 0.823; AUC_WH_ = 0.810). Besides, DeLong’s method indicated the AUC value of LR to be significantly lower than those of GBM, RF, and ANN for both the clinics (*p*-value < 0.05). For each ML algorithm under study, Table 3 provides the AUC values obtained using the testing dataset. The ML algorithms’ AUC values on the testing dataset are not significantly different from their cross-validation performance, thereby suggesting that the model is generalizable and not overfitting on the training data. Based on the results obtained from the hold-out sample and testing dataset, GBM is dominant in accurately classifying patients as punctual and unpunctual. 

The performance of the ML algorithms with respect to computational complexity is presented in Table 4, where the CPU times required to build each model on the training dataset for the ENT and WH clinics are summarized. For both clinics, LR is the fastest and achieves a CPU time that is several orders of magnitude smaller than the other three ML algorithms under study. GBM is the distant second with respect to computational complexity for both the clinics and is closely followed by ANN. RF performed the worst with regard to training time as it required 31.4% and 34.7% more CPU time than ANN for the ENT and WH clinics, respectively. However, the runtimes of the non-linear ML algorithms (RF, GBM, and ANN) are still tractable as they required less than 90 min for training, which is still practical for real-life applications. 

The third criterion for the model evaluation is interpretability. LR provides the highest interpretability as it identifies the key predictors (*p*-value < 0.05) and quantifies its impact on patient lateness using odds ratio (OR) (see Table 5 and Table 6). Among the continuous variables, lateness history has the highest impact on tardy arrivals for both the clinics under study. Besides, the number of clinic visits, marital status, weather conditions, and appointment time (morning vs. afternoon) are significantly associated with late arrivals. On the other hand, appointment duration is the only variable that has a contrasting impact on the two clinics. Compared with brief appointments (typically scheduled for 15 min), intermediate appointments are likely to be punctual for ENT clinics but late for WH clinics. It can also be observed that some of the significant variables are non-overlapping among the two clinics under study.

**Figure 3:**
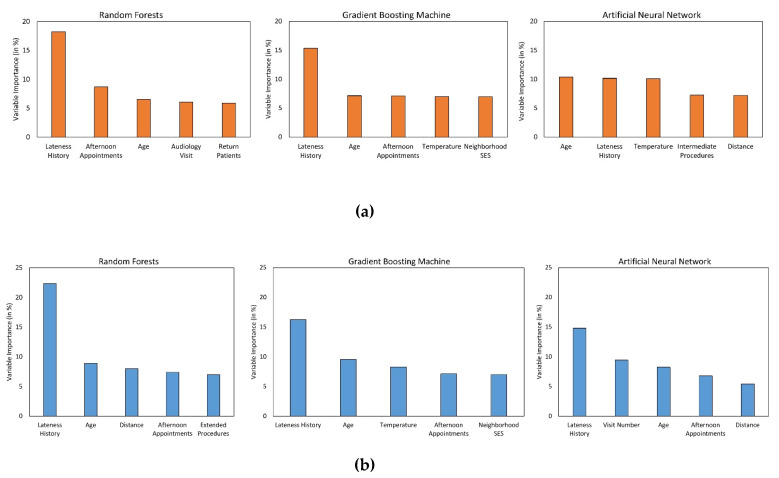
Plot of variable importance measure of the top 5 predictors associated with (**a**) ENT and (**b**) WH clinics.

While it is cumbersome to quantify the influence of each predictor on patient punctuality using the RF, GBM, or ANN algorithms, it is possible to estimate their relative importance in predicting the outcome class. Figure 3 visually illustrates the relative importance of the top five variables in predicting delayed arrivals in the ENT and WH clinics, respectively. The independent variables (*x*-axis) are listed in the decreasing order of their importance, while the *y*-axis shows the relative importance of each variable. Lateness history appears to be a powerful predictor of patient punctuality in both the clinics as it is the most crucial variable for all the ML algorithms except one, in which it is ranked second. Likewise, patient’s age and afternoon appointments are the next most important features since they are consistently rated among the top five variables for both the clinics. Moreover, it is also interesting to note that predictors such as forecasted temperature and distance were not significant for the LR algorithm but are ranked as critical predictors by at least two of the three non-linear ML algorithms. However, certain variables were found to be of high importance by only one of the three non-linear ML algorithms (e.g., neighborhood SES by GBM).

## 4. Discussion

Recent studies have leveraged ML models to enable patient-specific predictions and effective delivery of care [19,29,30,31,32]. While most of these prior studies focused on estimating the disease risk of individuals [33,34,35], some researchers have also focused on predicting clinical uncertainties such as no-shows and demand [36,37]. This study focuses on the latter category and seeks to predict the patient-specific risk of late arrivals at ambulatory care centers using ML algorithms. As opposed to previous studies on late arrivals, this research is among the first to consider unique features pertaining to the patient, appointment, and environment. Besides, the risk prediction models developed are evaluated across different specialties, unlike the traditional approach of relying on a single clinic. Overall, the analysis indicated that the information available in the EMR is sufficient to predict patient punctuality with high accuracy using advanced ML models. Therefore, hospitals and clinics can obtain valuable insights without spending substantial money on resources or establishing a new mechanism to collect the required data. Concordant with other study findings, ensemble ML algorithms (GBM and RF) are found to have a significantly better predictive performance for both the clinics considered in this research [19,24,38]. Besides, the relationship between the predictors and outcome appears to be non-linear as the linear mapping function of LR resulted in an inferior predictive performance as opposed to the ML algorithms capable of handling non-linearity (GBM, RF, and ANN). Nevertheless, a single ML algorithm could not perform the best with respect to all the three types of evaluation measures. While a linear algorithm such as LR is ideal for faster training and interpretable predictions, ML models capable of handling a non-linear decision boundary are best-suited to optimize the predictive performance. Therefore, the decision-maker has to make a trade-off between these criteria.

Very few studies have focused on identifying factors associated with patient unpunctuality [10]. In contrast, this research identified the main determinants of patient punctuality that are specific to each clinic and the ML algorithm. Consistent with the literature, age and appointment time were found to have a significant impact on late arrival [5,10]. Additionally, “Lateness History” was identified as the most critical variable for both the clinics. Contrary to the prior research, gender did not have a substantial influence in predicting late arrivals at the two clinics [5]. Besides, weather conditions and appointment day of the week are significantly associated with late arrivals for LR but did not affect the prediction accuracy for the other three ML algorithms. These findings would allow the clinics to strategically focus on critical predictors that can improve on-time arrivals instead of analyzing the exhaustive list of variables available in the EMR. As a result, it can potentially save time and unproductive labor in the long-run. Moreover, these variables can also help the administrators to determine the key performance metrics that must be included in the performance monitoring healthcare dashboards. For example, if morning appointments are likely to be late, then the percentage of late arrivals during that period would be a suitable metric to monitor and improve over time. Further, the clinic can adopt strategies to avoid late arrivals by understanding the key determinants. For instance, if thunderstorms are expected to increase late arrivals, then the clinic could consider different options to mitigate delays such as providing transportation assistance (e.g., information on local reliable transportation options, facilitating ride-share services), home healthcare, or virtual care (telehealth service).

An ML algorithm with good predictive performance would provide tremendous value to the clinic and healthcare practitioner as they can leverage them for targeted interventions and efficient planning [39,40]. In particular, instead of adopting appointment reminder strategies (e.g., phone calls, text messages) for all scheduled patients, the staff can only contact patients who are predicted to arrive late. Besides, the ML model can be used to improve on-time arrivals by evaluating the patient-specific risk of late arrival for all possible combinations of appointment day and time, and scheduling the patient to a slot in which he/she is likely to be on-time. Another approach to managing tardy arrivals is to integrate the patient specific-risk of late arrival into the appointment system design. For example, given that a patient is likely to arrive late, schedulers can strategically book the patient by overlapping his/her appointment time with a patient who is expected to come on time so that valuable clinic resources, such as doctors, are effectively utilized. For example, if a patient who is likely to be on-time is scheduled from 9 a.m. to 9:30 a.m., and if a patient who is expected to be late by five or more minutes calls for an appointment, then that patient can be scheduled for 9:25 a.m. instead of 9:30 a.m. to compensate for a delayed arrival. Note that the best overlapping position should be established based on extensive analytical and simulation experiments.

The study results and findings successfully demonstrate the ability of the ML model to accurately predict tardy arrivals well in advance, thereby indicating its suitability as a useful clinical decision support system (CDSS) for managing and mitigating patient unpunctuality. However, this research also has some limitations. Even though the data from two different clinics are utilized to develop the predictive model and analyze its results, the findings are still not generalizable across all ambulatory care centers. Therefore, future work should consider many other clinic types to identify the determinants that are common across all ambulatory settings and factors that are clinic-specific. Second, a patient is classified to be late if he/she arrives five minutes beyond the scheduled appointment time. While this cut-off is chosen based on the clinics under consideration, its value could change for other clinics. Certain clinics enforce a strict policy where a patient is considered to be late if he/she arrives beyond the scheduled appointment time [41,42]. In contrast, some clinics provide a grace period of 15 min before categorizing a patient to be late [43]. Therefore, the variable importance and predictive performance of the ML algorithm could change depending on the cut-off value. However, in this research, the benefit of using an ML algorithm for predicting late arrivals in the chosen clinic is established, and models developed for other clinics could adopt the proposed framework and use a cut-off that is suitable for their clinical setting. Third, the ML models developed in this research do not consider the possibility of patient no-show since it was rarely observed in the clinics under study. However, some clinics may experience substantial cases of both no-show and late arrivals [44]. In such situations, the ML models can be easily extended to incorporate three outcome classes: on-time arrival, late arrival, and no-show.

## 5. Conclusions

Clinics typically overbook appointments and operate close to their maximum capacity to handle the soaring demand for ambulatory services [45,46]. Under such circumstances, even a few late-arriving patients could disrupt the schedule and adversely affect the quality of care, doctor utilization, and patient satisfaction [47]. Therefore, in this research, four ML models (LR, RF, GBM, and ANN) to predict late arrivals are developed, and their evaluation supports the following conclusions. First, ML algorithms can enable a clinic to accurately detect late arrivals in advance using the information stored in the EMR. Second, tree-based ensembles (GBM and RF) consistently achieve a superior predictive performance, but a single ML algorithm could not perform best with respect to accuracy, training time, and interpretability. Third, the key predictors necessary to predict tardy arrivals are found to be model-specific. Fourth, late arrivals are likely to be affected by patient-, visit- and environmental-level predictors. Clinics can leverage the generic systematic approach presented in this research to develop a decision support system for targeted intervention and efficient planning, which can, in turn, improve the quality of care, resource utilization, and patient satisfaction. Moreover, the insights drawn from the analysis will enable healthcare administrators to manage and mitigate late arrivals. Finally, future research can also leverage the patient-specific lateness probability obtained from the ML model to design effective scheduling strategies.

## Figures and Tables

**Table 1:** Description of predictor variables.

Features	Variable Type	Source
**Patient-related**		
● Age (in years)	Continuous	Raw
● Gender	Categorical (Male, Female)	Raw
● Marital Status	Categorical (Single, Married, Divorced, Separated, Widowed)	Raw
● Race	Categorical (African American, Asian, Caucasian, Other)	Raw
● Visit Count	Continuous	Derived
● Insurance Group	Categorical (Medicare, Medicaid, Private, Uninsured)	Raw
● Patient Types	Categorical (New, Return)	Raw
● Commuting Distance (in miles)	Continuous	Derived
● Neighborhood SES	Continuous	Derived
**Appointment-related**		
● Month of the Year	Categorical (Jan, Feb, …, Dec)	Raw
● Day of the Week	Categorical (Mon, Tue, …, Fri)	Raw
● Appointment Time	Categorical (Morning, Afternoon)	Raw
● Procedure Duration	Categorical (Brief, Intermediate, Extended)	Raw
● Before National Holiday	Categorical (Yes, No)	Derived
● After National Holiday	Categorical (Yes, No)	Derived
● Lateness History	Continuous	Derived
**Clinic-related**		
● Resource Type	Categorical (MD, Nurse,….)	Raw
● Visit Type	Categorical (ENT, Audiology,…)	Raw
**Environment-related**		
● Temperature (in ^o^F)	Continuous	Derived
● Visibility (in miles)	Continuous	Derived
● Weather Condition	Categorical (fog, light rain, normal, thunderstorms, snow)	Derived

**Table 2:** Summary of predictor variables that are common for ear-nose-throat (ENT) and women’s health (WH) clinics.

Predictors	ENT Clinic		WH Clinic	
	On-time Arrival (*n* = 36,191)	Late Arrival (*n* = 10,230)	On-time Arrival (*n* = 59,965)	Late Arrival (*n* = 19,329)
**Gender**				
● Female	45.93%	46.87%	100%	100%
● Male	54.07%	53.13%	-	-
**Marital Status**				
● Divorced	4.42%	4.89%	5.53%	5.14%
● Married	21.63%	30.71%	60.39%	55.35%
● Separated	0.72%	0.81%	1.68%	2.06%
● Single	70.14%	59.42%	30.79%	36.38%
● Widowed	3.09%	4.17%	1.61%	1.07%
**Race**				
● African American	6.06%	8.98%	4.87%	8.29%
● Asian	2.06%	2.15%	1.85%	2.46%
● Caucasian	80.04%	73.48%	83.49%	75.86%
● Other	11.84%	15.39%	9.79%	13.39%
**Insurance Group**				
● Medicaid	31.13%	38.91%	25.31%	18.91%
● Medicare	22.68%	14.62%	4.75%	7.3%
● Private	44.54%	44.05%	68.08%	72.51%
● Uninsured	1.65%	2.42%	1.86%	1.28%
**Holiday Event**				
● Before National Holiday	1.66%	1.86%	1.81%	1.66%
● After National Holiday	3.94%	3.52%	4.52%	4.41%
**Patient Type**				
● New	27.74%	28.54%	19.07%	19.16%
● Return	72.26%	71.46%	80.93%	80.84%
**Procedure Duration**				
● Brief	35.29%	32.58%	35.29%	32.58%
● Extended	9.69%	11.26%	9.69%	11.26%
● Intermediate	55.02%	56.16%	55.02%	56.16%
**Appointment Day of Week**				
● Monday	20.44%	19.06%	20.46%	19.32%
● Tuesday	18.57%	17.14%	23.87%	24.26%
● Wednesday	22.652%	23.47%	16.15%	16.22%
● Thursday	21.67%	22.23%	21.14%	21.12%
● Friday	16.67%	18.1%	18.38%	19.08%
**Appointment Time**				
● Afternoon	46.38%	40%	42.57%	38.47%
● Morning	53.62%	60%	57.43%	61.53%
**Weather Conditions**				
● Fog	3.84%	3.77%	3.97%	3.93%
● Light Rain and Drizzle	5.8%	6.11%	6.15%	6.49%
● Normal	87.79%	87.49%	87.27%	86.92%
● Rain and Thunderstorms	1.33%	1.44%	1.33%	1.3%
● Snow	1.24%	1.19%	1.28%	1.36%
**Continuous Variables**				
Age	35.24 ± 28.89	28.04 ± 26.88	34.16 ± 11.62	36.73 ± 13.73
Lateness History	0.07 ± 0.16	0.73 ± 0.3	0.61 ± 0.3	0.11 ± 0.18
Neighborhood SES	0.115 ± 0.35	0.1018 ± 0.36	0.1 ± 0.37	0.11 ± 0.35
Distance	29.42 ± 38	28.81 ± 36.49	19.9 ± 50.84	20.63 ± 44.54
Temperature	57.04 ± 19.7	58.06 ± 19.74	58.09 ± 19.69	57.6 ± 19.56
Visibility (MPH)	8.82 ± 2.54	8.86 ± 2.5	8.82 ± 2.52	8.81 ± 2.53

**Table 3:** AUC value based on testing datasets for the ENT and WH clinics.

ML Algorithm	ENT Clinic	WH Clinic
Logistic Regression	0.828	0.781
Random Forests	0.868	0.849
Gradient Boosting Machines	0.904	0.863
Artificial Neural Network	0.861	0.837

**Table 4:** Training time of different ML algorithms.

ML Algorithm	Computational Time (in seconds)	
	ENT Clinic	WH Clinic
Logistic Regression	8.55	13.70
Random Forests	1447.49	4392.15
Gradient Boosting Machines	1001.32	3082.68
Artificial Neural Network	1101.53	3260.67

**Table 5:** Significant predictors of the ENT clinic and their odds ratios in the logistic regression model.

Variable (Reference)	Odds Ratio	Odds Ratio 95% CI	*p*-Value
Visit	1.08	(1.07, 1.09)	< 0.0001
Lateness History	19.24	(15.48, 23.9)	< 0.0001
Visibility	0.97	(0.95, 0.99)	0.0139
Marital Status (Married)			
● Divorced	0.78	(0.61, 0.98)	0.0342
● Single	0.86	(0.72, 1.02)	0.0460
After National Holiday	0.78	(0.6, 1.01)	0.0484
Resource Type (MD)			
● Audiologist Assistant	2.75	(1.89, 4.01)	0.0000
● Audiologist	2.28	(1.52, 3.43)	0.0001
● Other	2.41	(1.76, 3.3)	0.0000
● Physician Assistant	1.17	(0.99, 1.39)	0.0480
● Speech Pathologist	1.80	(1.32, 2.46)	0.0002
Visit Type (Pediatric Surgery)			
● Audiology	0.65	(0.47, 0.9)	0.0093
● ENT	0.76	(0.61, 0.95)	0.0171
Procedure Duration (Brief)			
● Extended	0.57	(0.44, 0.73)	0.0000
● Intermediate	0.80	(0.69, 0.93)	0.0037
Appointment Time (Morning)			
● Afternoon	0.62	(0.56, 0.68)	< 0.0001
Appointment Weekday (Monday)			
● Thursday	0.84	(0.72, 0.98)	0.0225
Weather Conditions (Normal)			
● Thunderstorm	1.43	(0.96, 2.12)	0.0358
● Snow	1.59	(1, 2.53)	0.0483

**Table 6:** Significant predictors of the WH clinic and their odds ratios in the logistic regression model.

Variable (Reference)	Odds Ratio	Odds Ratio 95% CI	*p*-Value
Visit	1.04	(1.03, 1.05)	< 0.0001
Lateness History	4.81	(4.21, 5.49)	< 0.0001
Age	0.99	(0.98, 1.00)	0.0002
Marital Status (Married)			
● Single	0.93	(0.86, 0.99)	0.0283
Resource Type (MD)			
● Nurse	1.96	(1.21, 3.18)	0.0060
Visit Type (Gynecology)			
● Obstetrics	1.28	(1.18, 1.4)	< 0.0001
● Gynecologic Surgery	1.32	(1.17, 1.48)	< 0.0001
● Urogynecology	1.88	(0.90, 3.92)	0.0412
● Maternal-fetal Medicine	1.37	(1.19, 1.59)	< 0.0001
● Endocrinology	1.25	(1.08, 1.44)	0.0023
● Radiology	1.61	(1.42, 1.81)	< 0.0001
Patient Type (New)			
● Return	1.41	(1.25, 1.60)	< 0.0001
Appointment Duration (Brief)			
● Intermediate	1.18	(1.08, 1.29)	0.0002
Appointment Time (Morning)			
● Afternoon	0.8	(0.75, 0.85)	< 0.0001
Weather Conditions (Normal)			
● Thunderstorm	1.16	(1.02, 1.31)	0.0235
